# Fibrinolytic Serine Proteases, Therapeutic Serpins and Inflammation: Fire Dancers and Firestorms

**DOI:** 10.3389/fcvm.2021.648947

**Published:** 2021-03-25

**Authors:** Jordan R. Yaron, Liqiang Zhang, Qiuyun Guo, Shelley E. Haydel, Alexandra R. Lucas

**Affiliations:** ^1^Center for Personalized Diagnostics and Center for Immunotherapy, Vaccines and Virotherapy, The Biodesign Institute, Arizona State University, Tempe, AZ, United States; ^2^School for Engineering of Matter, Transport and Energy, Ira A. Fulton Schools of Engineering, Arizona State University, Tempe, AZ, United States; ^3^Center for Bioelectronics and Biosensors, The Biodesign Institute, Arizona State University, Tempe, AZ, United States; ^4^School of Life Sciences, Arizona State University, Tempe, AZ, United States

**Keywords:** serpin, thrombolysis, fibrinolysis, coagulation, inflammation, serine protease, infection, virus

## Abstract

The making and breaking of clots orchestrated by the thrombotic and thrombolytic serine protease cascades are critical determinants of morbidity and mortality during infection and with vascular or tissue injury. Both the clot forming (thrombotic) and the clot dissolving (thrombolytic or fibrinolytic) cascades are composed of a highly sensitive and complex relationship of sequentially activated serine proteases and their regulatory inhibitors in the circulating blood. The proteases and inhibitors interact continuously throughout all branches of the cardiovascular system in the human body, representing one of the most abundant groups of proteins in the blood. There is an intricate interaction of the coagulation cascades with endothelial cell surface receptors lining the vascular tree, circulating immune cells, platelets and connective tissue encasing the arterial layers. Beyond their role in control of bleeding and clotting, the thrombotic and thrombolytic cascades initiate immune cell responses, representing a front line, “off-the-shelf” system for inducing inflammatory responses. These hemostatic pathways are one of the first response systems after injury with the fibrinolytic cascade being one of the earliest to evolve in primordial immune responses. An equally important contributor and parallel ancient component of these thrombotic and thrombolytic serine protease cascades are the *ser*ine *p*rotease *in*hibitors, termed *serpins*. Serpins are metastable suicide inhibitors with ubiquitous roles in coagulation and fibrinolysis as well as multiple central regulatory pathways throughout the body. Serpins are now known to also modulate the immune response, either via control of thrombotic and thrombolytic cascades or via direct effects on cellular phenotypes, among many other functions. Here we review the co-evolution of the thrombolytic cascade and the immune response in disease and in treatment. We will focus on the relevance of these recent advances in the context of the ongoing COVID-19 pandemic. SARS-CoV-2 is a “respiratory” coronavirus that causes extensive cardiovascular pathogenesis, with microthrombi throughout the vascular tree, resulting in severe and potentially fatal coagulopathies.

## Introduction

Hemostatic control of bleeding by clot formation (thrombosis) and the subsequent dissolution through fibrinolysis (also termed thrombolysis) are essential components in the front line response to trauma (1). In the past decade, intensive research has revealed that thrombosis and fibrinolysis are extensively involved in immune pathologies not directly linked to clotting or hemorrhage, including disorders related to sterile inflammatory diseases and microbial and viral infections (2). Interactions between coagulation pathways and the inflammatory immune response are now known to be essential to maintaining health and limiting disease.

The interaction between coagulation and inflammation is bidirectional, a “two-way street,” and one begets the other (3). Coagulation is used here to refer to thrombosis (clot formation) and thrombolysis (clot breakdown). It is well-understood that unregulated clotting or bleeding can have severe adverse consequences. Too much clotting causes occlusion of circulating blood (e.g., blocking the circulation), while too much fibrinolysis leads to hemorrhage and blood loss, and an excess of either can be fatal. To further complicate this interaction, in some cases severe vascular damage or infection causes excess thrombosis, consumption of clotting factors and eventual deficit in the homeostatic balance and excess fibrinolysis. This consumptive coagulopathy (CC) or disseminated intravascular coagulation (DIC) markedly increases mortality in viral and bacterial sepsis. Unregulated inflammation also causes severe tissue disruption, endothelial damage, microthrombotic occlusions, vascular leakage, hemorrhage, and shock with death. Elucidation of the cross-talk of these pathways (termed “thromboinflammation”) makes eminently clear that inflammation can cause clotting, and clotting can cause inflammation, thus the regulation of one pathway affects the other (4).

Serine proteases are highly active and one of the most prevalent protease classes, driving the thrombotic and thrombolytic cascades. Dysregulation of these coagulation pathway proteases leads to onset and/or exacerbation of numerous diseases, including rare bleeding disorders, chronic lung disease, septic shock (whether viral, bacterial, or fungal in origin), DIC (or CC), and neurodegeneration. The thrombotic and thrombolytic cascades are intrinsically regulated by the *ser*ine *p*rotease *in*hibitor (*serpin*) superfamily (5). Recent investigations have identified numerous immune modulating functions for serpins, clearly demonstrating that these complex inhibitors directly interact with and influence immune cell responses and regulate inflammation beyond direct effects on the thrombotic and thrombolytic pathways (6). Thus, a complete understanding of the role of the thrombotic and thrombolytic proteases, and the serpins that regulate their function in the circulating blood, may lead to novel therapeutic avenues for treating a diverse array of immunopathologies.

The role of the thrombotic pathway in inflammation has been extensively highlighted in numerous reviews (4, 7–10). In this review, we will discuss the interaction of the fibrinolytic pathway with inflammatory responses and the bidirectional regulation of these responses, both fibrinolytic and inflammatory, by serpins. We begin with an exploration of the evolutionary roots of the coagulation-associated pathways, both thrombotic and thrombolytic, and the serpins that regulate these pathways, as evidence for their origins in primordial immune responses. We then focus specifically on the fibrinolytic/thrombolytic pathway, the interaction of serine protease activity in fibrinolysis and inflammation, and their contributing roles in disease. Next, we discuss the evidence for utilizing serpins as therapeutics designed to modulate the fibrinolytic response in disease. We will conclude with a brief discussion of the fibrinolytic pathway and serpins in the pathogenesis (and potentially treatment) of the ongoing COVID-19 pandemic caused by SARS-CoV-2.

## Immune Origins at the Ancient Roots of Coagulation Proteases and Serpins

### Thrombosis and Thrombolysis–Evolutionary Roots

**Figure 1:**
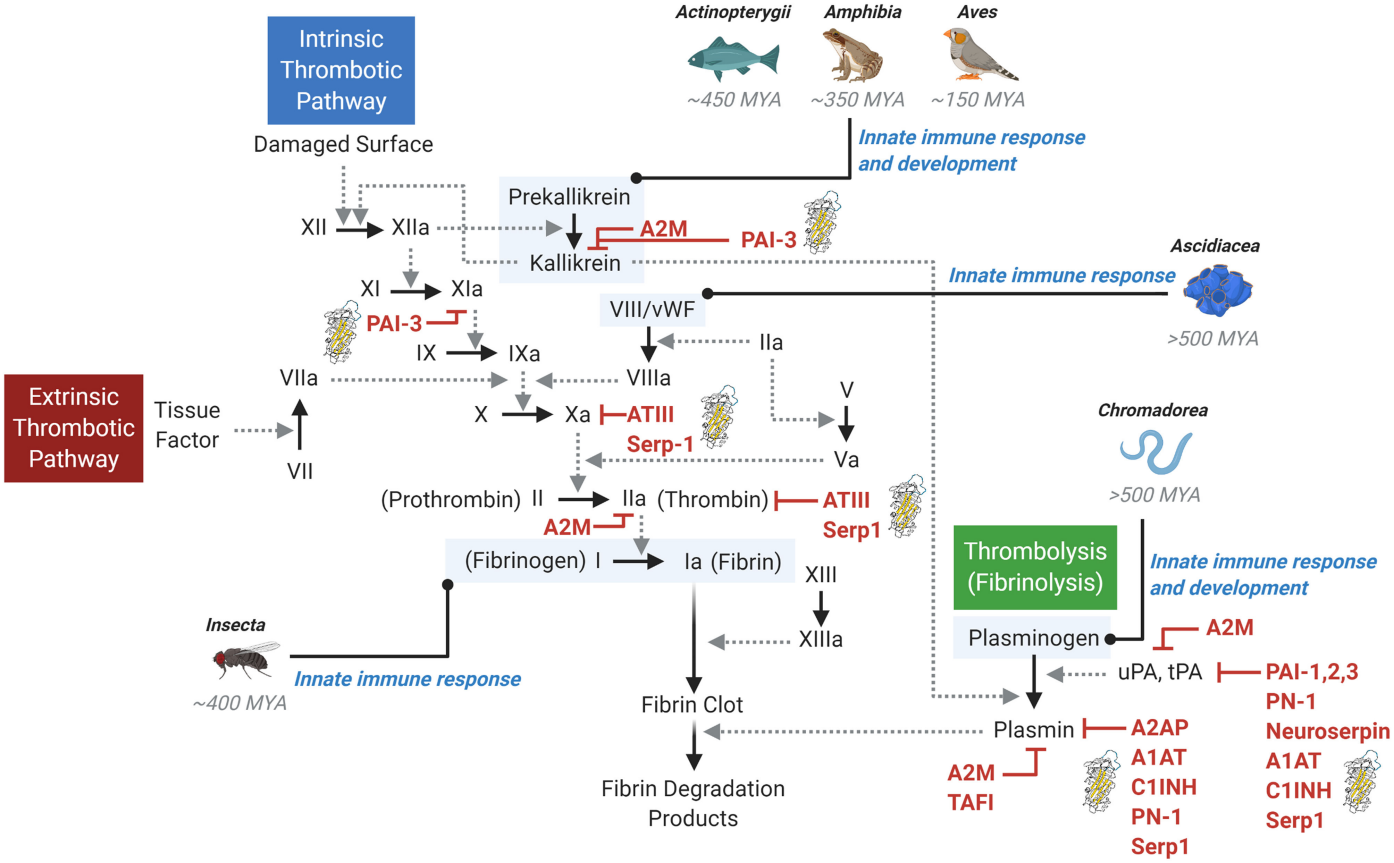
The thrombotic and thrombolytic cascades and primordial immune response. The thrombotic pathways (intrinsic and extrinsic) and the thrombolytic (fibrinolysis) pathway involve a complex cascade of protease activation. Solid arrows indicate the conversion to an active protease, while dotted line arrows indicate the activity of the activating upstream protease. A variety of inhibitors are shown, with serpin inhibitors denoted by a serpin protein structural image. Examples of early primordial immune response origins are noted in context of the pathways. MYA, million years ago.

Serine proteases involved in coagulation are functionally conserved across the Kingdom *Animalia* and represent an ancient class of proteins. Emerging evidence suggests that independent evolution has occurred for at least two separate functions for these pathways: (i) control of thrombotic/thrombolytic responses and (ii) regulation of the immune response. While some pro-thrombotic (clotting) enzymes appear to have emerged as early as 700 million years ago, the genes and proteins required for the conversion of fibrinogen to fibrin did not appear until 500–600 million years ago (11). This timeline appears to have coincided with the emergence of fibrinolysis 570 million years ago (in the Precambrian period) (12). While much has been discovered in the developmental biology-based study of clotting across *Animalia*, the clotting “toolkit” has been found to differ greatly amongst animals. Exploration of the ancient roots of clotting now reveals that the coagulation pathways may originally have had central roles in innate immune responses, or inflammation (Figure 1).

Mammals (Class *Mammalia*) harbor the most complex coagulation system, with the classically defined contact activation or “intrinsic” pathway and the tissue factor/factor VII system or the “extrinsic” pathway (13, 14). The pathways converge, resulting in a complex downstream cascade of protease activation events leading to the activation of factor X and thrombin, conversion of fibrinogen to fibrin, and ultimate generation of a stable fibrin clot (Figure 1). In the vascular system, thrombi form on the surface of activated platelets, damaged endothelium in the lining of the arterial wall, and activated macrophage cells adherent to damaged endothelium. This entire interactive complex both activates and is activated by the kallikrein-kinin system. While apparently important to the activation of thrombosis, deficiency of this pathway affects thrombosis and modifies immune responses.

A careful examination of other animals reveals a distinct role for coagulation in the immune response. For example, despite the presence of components of the kallikrein-kinin system in birds (Class *Aves*), amphibians (Class *Amphibia*) and fish (Class *Actinopterygii*), evidence suggests these components do not drive clot formation, but rather, regulate angiogenesis and the immune response system (Figure 1). The process of clotting in these Classes is regulated by the extrinsic tissue factor-directed pathway (15). Looking further, lower-level animals (e.g., invertebrates) also contain mechanisms for regulating clotting. Due to their open circulatory system and propensity for massive loss of hemolymph (the equivalent of combined blood and lymphatic fluid), clotting evolved very efficiently in insects (Class *Insecta*) and is central to innate immunity in *Drosophila* (16). Class *Ascidiacea*, which include sac-like marine invertebrate filter feeders, is among the most ancient coagulation systems investigated. While their plasma contains some blood clotting components (such as von Willebrand factor, vWF), blood in *Ascidacea* animals does not clot, and these components are predominantly used to regulate innate immune responses (Figure 1) (17, 18).

A similar primordial role in innate immunity can be found for fibrinolysis, a cascade that balances and is complementary to the coagulation cascade which is responsible for dissolution of a fibrin clot. In *Mammalia*, fibrinolysis is initiated by the conversion of inactive, circulating plasminogen into active plasmin by serine proteases referred to as the plasminogen activators, tissue- and urokinase-type plasminogen activators (tPA and uPA, respectively) (Figure 1). Activated plasmin then breaks down cross-linked fibrin, resulting in dissolution of the thrombus. Investigations into lower-level organisms distinctly reveal the immune response origins of the thrombolysis pathway. For example, *Caenorhabditis elegans* (Class *Chromadorea*), despite its lack of vasculature, blood, or hemolymph, expresses a functional plasminogen-like protease required for organ development and innate immunity (19, 20). Thus, fibrinolysis represents another component of the primordial innate immune response that has been preserved for millions of years.

### Serpins–Evolutionary Roots

**Figure 2:**
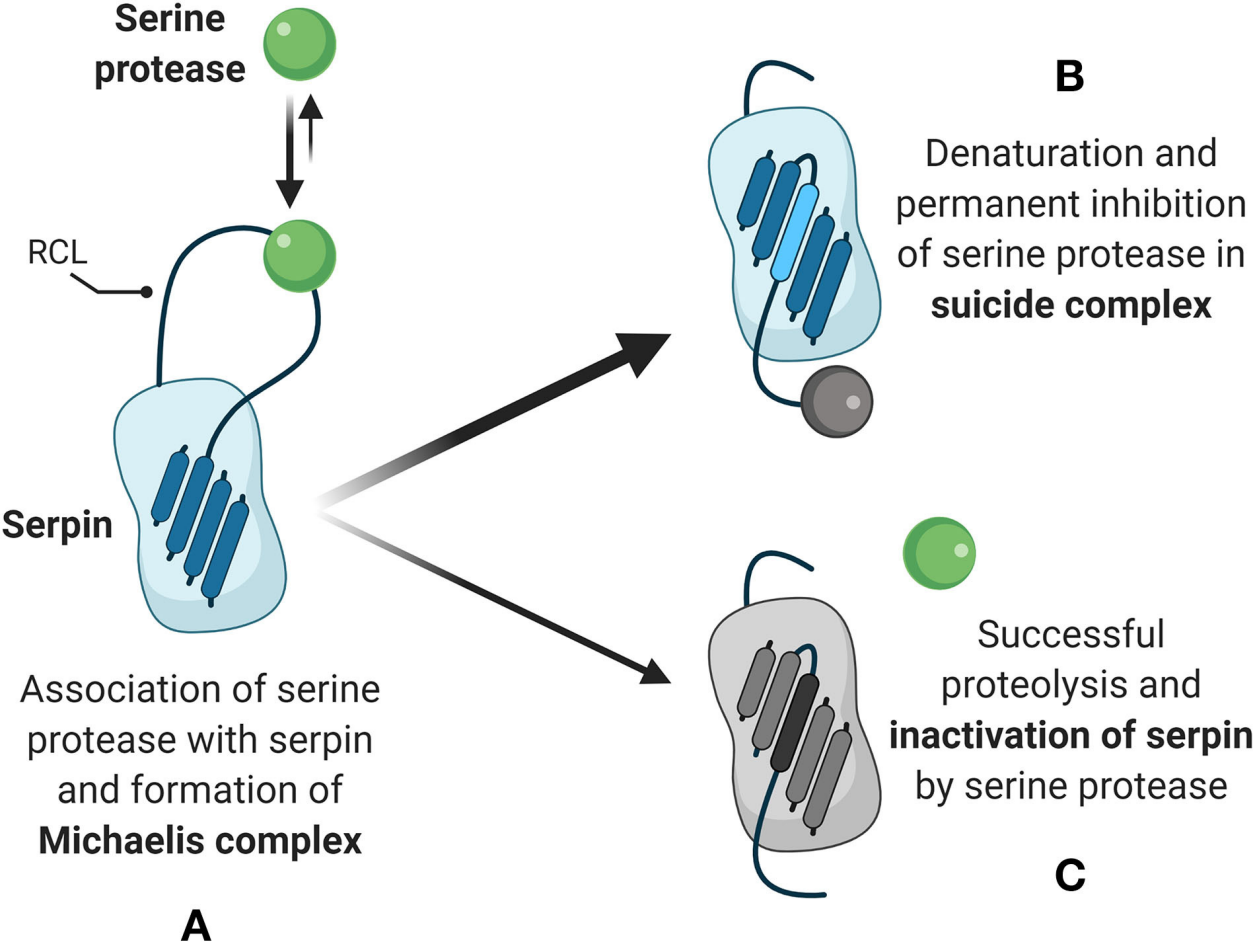
Serpin-dependent mechanism of protease inhibition. **(A)** Natively folded serpins present a reactive center loop (RCL) which acts as a bait for interaction with active serine proteases. Upon association, initiation of proteolytic activity by the protease triggers the formation of a Michaelis complex and covalent bonding of the serpin with the protease. **(B)** The primary outcome of Michaelis complex formation is reconfiguration of the serpin, with the RCL inserting as the third of five strands in the core beta sheet and permanent denaturation of the target serine protease. **(C)** The secondary and less frequent outcome is completion of proteolytic activity and dissociation of the active serine protease, while the RCL continues to insert as the third of five strands in the serpin core beta sheet.

As counterparts to the proteases that mediate fibrinolysis, serpins are the equally-ancient, intrinsic protease inhibitors that arose in *Animalia* some 650–700 million years ago. Serpins are highly metastable proteins characterized by two key structural components: a reactive center loop (RCL) and a 4-stranded, core beta-sheet (termed the “A” beta-sheet). The reactive center loop contains a protease recognition sequence which acts as a bait for activated serine proteases (Figure 2). Cleavage of the protease recognition sequence by the appropriate protease creates a transient Michaelis complex, as occurs in any generalized enzyme-substrate interaction, where the protease and the serpin are covalently bonded at the active site of the protease (Figure 2). Upon cleavage of the recognition site, the metastability inherent in the serpin is released and the serpin-protease pair initiates a dramatic (on a molecular scale) conformational change (Figure 2). The reactive center loop “swings” 70 angstroms across the protein and inserts itself as the third strand in a now five-stranded beta sheet. The conformational change of the serpin induces a deformation of the active site in the protease, disallows completion of the protease-substrate interaction, and permanently denatures both the serpin and protease in what is referred to as a “suicide complex” (Figure 2). Because serpins are tuned to their inhibitor by changes in the protease recognition sequence in their RCL, multiple gene duplications have resulted in the ability for serpins to become tailored to a wide variety of proteases. Interestingly, in some cases individual serpins have lost their canonical protease inhibitory function, as in the case of Maspin, which does not form suicide complexes upon protease digestion with any tested protease and which is associated with tumor suppression by a mechanism that is still poorly defined (21). Accordingly, the genomes of most members of *Animalia* contain a multitude of serpins with human and mouse genomes encoding 37 and 60 serpins, respectively (22, 23). At the other end of the evolutionary spectrum, the genome of *Caenorhabditis elegans* encodes nine serpins (24).

The evolutionary origins of immune regulation by serpins are exemplified by *Drosophila*. Persephone is a circulating serine protease in *Drosophila* upstream of the fly Toll pattern recognition receptor pathway and activates the protease Spätzle (25). Deficiency in the Spn43Ac serpin, which regulates Persephone, leads to constitutive activation of Spätzle in the *Drosophila* innate immune response and results in developmental lethality (the *nec* phenotype) (25). Thus, both serine proteases and their serpins have evolutionary roots in regulating inflammation and the innate immune response.

## Overview of the Fibrinolytic Machinery

### Serine Proteases of the Fibrinolysis System

**Figure 3:**
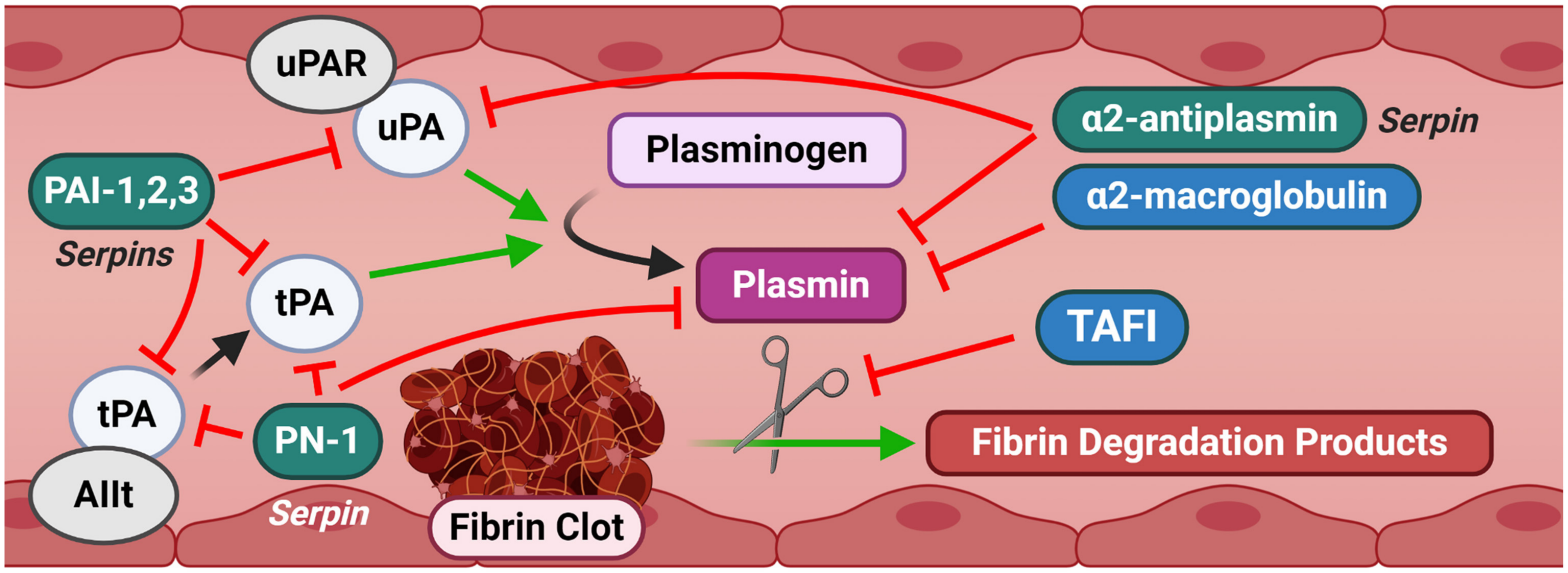
Canonical signaling of the fibrinolysis pathway. Fibrinolysis is characterized by the degradation of a fibrin clot into degradation products by plasmin. Plasmin is generated from plasminogen by uPA and tPA. Several serpins and other inhibitors provide a tight regulation of this cascade. Substantial promiscuity exists across multiple elements of the pathway, providing redundant controls against inappropriate activation. AIIt and uPAR are shown as representative canonical fibrinolytic receptors for brevity. AIIt, Annexin II tetramer; PAI-1,2,3, Plasminogen Activator Inhibitor-1, 2, 3; PN-1, Protease Nexin-1; TAFI, Thrombin activatable fibrinolysis inhibitor; tPA, Tissue-type plasminogen activator; uPA, Urokinase-type plasminogen activator; uPAR, Urokinase-type plasminogen activator receptor.

Fibrinolysis is ultimately mediated by the activity of the serine protease plasmin (Figure 3). Initiation of fibrinolysis requires the conversion of the inactive zymogen plasminogen to active plasmin, which then subsequently breaks down cross-linked clots (Figure 3). Plasminogen is predominantly synthesized in the liver and secreted at a plasma concentration of ~150 μg/mL (26). Plasminogen is converted by plasmin primarily by the activity of two plasminogen activators, tPA and uPA, as discussed in the next paragraph. Forward feedback of plasmin activity on its own activators results in increased processing of plasminogen to accelerate the generation of additional active plasmin (27). Plasmin activation occurs *in situ* when plasminogen co-localizes with its activators in a “ternary complex” at C-terminal lysine residue binding sites on fibrin (28). Active plasmin then directly cleaves the cross-linked fibrin to dissociate the clot. Plasminogen coordination at the cell membrane, and therefore localized generation of plasmin at cell surfaces, is mediated by a number of receptors, with the predominant plasminogen receptor being Plg-R_KT_ (29, 30). Plg-R_KT_ is expressed by a wide variety of cell types in all tissues, including migrating immune cells, and co-localizes with the receptor for uPA (discussed below) (29). The expression of Plg-R_KT_ enhanced plasminogen activation by more than 12-fold, in part by coordinating its localization with uPA and tPA at the cell surface (29).

The serine proteases tissue- and urokinase-type plasminogen activators, tPA and uPA, respectively, are the key components of the plasminogen activation system. While tPA and uPA share only about 40% amino acid similarity, their basic structure is highly similar (31). The basal circulating levels of tPA and uPA are low compared to other circulating proteins, with tPA reported in the range of ~1–10 ng/mL and uPA at 2–10x lower levels (~0.1–0.3 ng/mL) (32–36). Synthesis of tPA occurs in abundance in both the vasculature and the central nervous system. In the nervous system, tPA is synthesized and released by neurons and glial cells and is constitutively active in a number of regions of the brain where its activity has been associated with neural plasticity (37). tPA has been identified in secretary vesicles after membrane depolarization and is rapidly localized to neuronal synapses (38). Further studies have identified that the activity of tPA is essential for the late phase of long-term potentiation and is a driver of synaptic growth (39). In the vasculature, synthesis of tPA occurs predominantly in endothelial cells and is stored in granules called regulated secretory organelles (RSOs). *In vitro* and *in vivo* experiments indicate that RSOs are trafficked and secreted rapidly in response to diverse physiologic stimuli, such as histamine (40), activated thrombin (41) and bradykinin, a metabolic product of the kinin-kallikrein system (42). While secreted tPA is active in the open circulation, its plasmin generation activity is enhanced by interaction with its receptor, the annexin A2 heterotetramer (AIIt), composed of two units of annexin A2 bound by a dimer of S100A10 (43). Binding of tPA to AIIt enhanced plasmin generation by 77-fold and mice deficient in S100A10, and therefore deficient in functional AIIt receptors, have 40% reductions in plasmin generation and reduced clearance of batroxobin-induced vascular thrombi (43, 44).

In contrast to the activity of tPA, uPA functions predominantly in innate, inflammatory immune responses and the tissue responses to injury, rather than the coagulation response (45). uPA is synthesized in a wide variety of tissues and cell types including vascular endothelial cells (46), hepatocytes (47), keratinocytes (48), renal tubular epithelial cells (49), neurons (50), and immune cells of both monocytic (51) and lymphocytic (52) lineages. Basal release of uPA is in the form of a single-chain zymogen with little or no proteolytic activity. uPA is further processed to a double-chain active enzyme that has a several hundred-fold higher activity by plasmin (53), kallikrein (54), activated factor XII (54), or trypsin-like proteases, for example, as released from tumors (55). While uPA can act in the open circulation, its enzymatic efficiency substantially increases by interaction with the urokinase-type plasminogen activator receptor (uPAR, also called CD87) (56). Interestingly, the activity of uPA itself is partially lowered by interaction with uPAR, but complex local coordination of uPA and its substrates (e.g., plasminogen) results in a net total increase in processivity by means of concentration and spacial protein-protein coordination effects (57). Interestingly, uPAR interacts promiscuously with components of the kallikrein pathway, FXII and AIIt, suggesting a broader, yet unexplored, role in regulating fibrinolytic and immune responses (43, 58, 59).

In addition to their function in fibrinolysis, plasmin, tPA, uPA and their receptors—Plg-Rs, the LDL receptor-related protein-1 (LRP1), and uPA receptor (uPAR), respectively,—have important functions in tissue remodeling and cell invasion. These thrombolytic proteases alter the extracellular matrix and modify cellular phenotype conversion via induction of intracellular signaling cascades. Thus far there have been 12 Plg-Rs identified, some of which are expressed on the cell surface and others are localized intracellularly (60). The dominant Plg-R expressed by macrophages is the plasminogen receptor Plg-R_KT_, which is a surface expressed receptor where plasminogen binds and is activated (61). The activation of plasminogen when bound to the macrophage-expressed receptor is required for efficient invasion and clearance of dead cells (62, 63). uPAR is moderately expressed in most tissues in a healthy organism, including the lungs, kidneys, spleen, blood vessels, uterus, bladder, thymus, heart, liver, and testes (64). However, uPAR expression is strongest in tissue actively undergoing extensive remodeling (65). For example, keratinocytes at the migrating edge of cutaneous wounds exhibit potent upregulation of uPAR and wounds heal poorly in uPAR-deficient mice (66, 67). Upon immunological activation, neutrophils, monocytes and T cells markedly upregulate uPAR expression (68–70). Exposure of uPAR to uPA enhances the differentiation of monocytes to macrophages (71). Expression of uPAR also dictates the interaction of macrophages and neutrophils in efferocytosis (the clearance of apoptotic cells), as well as phagocytosis of viable cells by macrophages. Deficiency of uPAR in either macrophages or viable neutrophils enhances phagocytosis, but deficiency of uPAR in both cell types blocks phagocytosis (72). Independent of its fibrinolytic activity, recombinant uPA elicits an anti-apoptotic response in cultured endothelial cells by specific induction of the X-linked inhibitor of apoptosis (XIAP) protein (73). Similarly, uPA attenuates macrophage apoptosis induced by Ox-LDL and ER stress by activation of ERK1/2 and downregulation of Bim (71). Likewise, recombinant tPA dose-dependently rescued cultured neurons from serum deprivation-induced apoptosis via a mechanism involving the PI-3 kinase pathway (74). Thus, serine proteases of the fibrinolytic cascade have essential fibrinolysis-independent functions in tissues and the immune system.

### Regulators of Fibrinolysis in Mammals

Serpins have evolved into a large class of regulatory proteins with extensive functions throughout circulating hematological and immune pathways in a wide range of organ systems from the cardiovascular tree to endocrine and neurological organs (75). As our knowledge of serpin sequences and structures have progressed, it is evident that widespread exchanges and combinations enabled serpins to target more than one pathway. As serpin functionality has evolved, some serpins retained immune regulating functions while concurrently expanding to target the thrombolytic cascades. In this section, we will discuss the known regulators of the thrombolytic/fibrinolytic cascades and in subsequent sections discuss their additional capacity to cross interaction and regulate immune and inflammatory responses with potential for providing new therapeutic reagents.

Serpin-dependent regulation of the fibrinolytic serine proteases in mammals is mediated by the plasminogen activator inhibitor (PAI)-1 (SERPINE1), PAI-2 (SERPINB2), and PAI-3 (SERPINA5; also called Protein C inhibitor, PCI) and protease nexin-1 (PN-1 or SERPINE2) against uPA and tPA, and by alpha-2-antiplasmin (SERPINF2) against activated plasmin. tPA is also regulated by a central nervous system-specific serpin, neuroserpin (SERPINI1). Two other serpins, alpha-1-antitrypsin (AAT or A1AT, SERPINA1) and Complement C1 inhibitor (C1INH, SERPING1), target fibrinolytic proteases but are better known for inhibition of other protease and inflammatory systems outside of the coagulation pathways will be briefly discussed.

Classical serpin-mediated inhibition of fibrinolytic serine proteases leads to permanent inactivation of both the serpin and protease via formation of a classic suicide complex (described above). Thus, tight control of fibrinolysis requires that circulating serpins are present in molar excess, or pre-synthesized and rapidly released from stores, without the need for transcription and translation. Accordingly, serpins account for up to 10% of circulating proteins in the circulation (76).

PAI-1, the principal member of the PAI protein family, is primarily produced by hepatocytes and secreted into the circulation by the liver. To a lesser extent, PAI-1 is synthesized and secreted by the kidney, spleen, heart, lung and adipose tissues (77). Additionally, circulating platelets continuously synthesize PAI-1, which is actively and rapidly released upon platelet activation and contributes to the stability of clots by limiting fibrinolysis (78). Circulating PAI-1 is usually present in a concentration range of 20–30 ng/mL, which is in three-fold excess of basal circulating tPA and up to 300-fold excess of basal circulating uPA (79). PAI-2 expression is restricted to keratinocytes, macrophages, activated monocytes, the placenta, and adipocytes (80). Circulating PAI-2 in healthy individuals is essentially undetectable, but drastically increases in pregnancy to over 250 ng/mL and rapidly declines postpartum (81). The skin is a major site for PAI-2 expression, where PAI-2 cross-links to the cornified cell membrane via transglutaminase during the terminal differentiation of keratinocytes to inhibit over-proliferation (82, 83). PAI-2 has been called the “undecided serpin” because its specific endogenous biological role has remained elusive, despite associations with regulating fibrinolysis and inflammation (84–86). PAI-1 is an efficient inhibitor of both uPA and tPA (2 × 10^7^ M^−1^ s^−1^ each) while PAI-2 effectively inhibits uPA (2 × 10^6^ M^−1^ s^−1^) and is a poor inhibitor of tPA (2 × 10^5^ M^−1^ s^−1^) (87, 88). PCI is predominantly an inhibitor of proteases in the thrombotic pathway, but detection of kidney-derived PCI complexed with uPA in the urine resulted in its identification as the third member of the PAI family as PAI-3 (89). Further justification for designating PCI as PAI-3 is the ability to inhibit plasma kallikrein and activated Factor XI (90), which are alternative activators of plasminogen (91, 92). Subsequent discussion of the roles of PAI serpins in disease will focus on PAI-1, as it is the principal serpin inhibitor of the fibrinolytic cascade.

Protease nexin-1 (PN-1 or SERPINE2) is expressed in diverse tissues during development, including cartilage, lung, skin, the urogenital tract and the nervous system, where it was originally identified as Glia-derived nexin (93). PN-1 is nearly undetectable in circulating plasma, amounting to ~1 ng/mL or 20 picomolar amounts (94). In contrast to the low levels in circulation, PN-1 is endogenously synthesized and stored in abundance in the alpha-secretory granules of platelets, from which it is rapidly released upon platelet activation (95). As the second-order rate constant of PN-1-tPA interaction is three orders of magnitude lower than PAI-1-tPA, it was expected that PN-1 is not the primary inhibitor of fibrinolysis (96). However, systematic *in vivo* studies by Boulaftali et al. demonstrated that PN-1 inhibits both fibrin-bound tPA and auto-activation of plasminogen by fibrin-bound plasmin and is an important regulator of fibrinolysis (97).

Alpha-2-antiplasmin (SERPINF2) is the major serpin inhibitor of activated plasmin. Alpha-2-antiplasmin is synthesized in the liver and kidney at nearly equivalent levels (98). Alpha-2-antiplasmin is present at significantly higher concentrations than the PAI serpins, with circulating levels at 70 μg/mL (99, 100). Thus, alpha-2-antiplasmin, in coordination with the PAI family and PN-1, mediate the multi-stage, tightly controlled serpin-dependent regulation of the fibrinolytic proteases.

Neuroserpin (SERPINI1) is expressed predominantly in the central nervous system and was originally isolated from chicken ventral spinal cord neurons (101). Neuroserpin, a highly specific inhibitor of tPA, is expressed from the growth cone of neurons and is a poor inhibitor of uPA and plasmin (102). Neuroserpin is found at a concentration of ~7.4 μg/L in the cerebrospinal fluid (CSF) and is significantly elevated in the CSF of patients with Alzheimer's Disease (103). Indeed, numerous neuropathologies are associated with dysregulation of neuroserpin. For example, familial encephalopathies with neuroserpin inclusion bodies are associated with mutations such as S49P-Syracuse (late onset encephalopathy) and S52R-Portland (early onset encephalopathy) (104). Polymers of misfolded neuroserpin stimulate inflammation via NF-kappaB and oxidative stress signaling in an unfolded protein response-independent manner and may contribute to neurodegeneration (105, 106). The spatiotemporal patterning of neuroserpin suggests a role in neuronal development and synaptogenesis via homeostatic maintenance of tissue by limiting excess tPA activity which can lead to cerebral ischemia and epilepsy (104). The critical and sensitive role of neuroserpin in regulating tPA activity outside of the circulating blood highlights the importance of effective control of these serine proteases in diverse tissues.

C1INH and A1AT act primarily as regulators of complement and neutrophil elastase, and mutations in both of these serpins cause severe genetic disorders (107, 108). C1INH deficiency causes angiogenic edema which can be life threatening and A1AT deficiency resulting from aggregating mutations which deplete circulating levels of A1AT causes severe lung damage and emphysema. Both serpins are believed to primarily regulate serine proteases outside of the coagulation cascades and have established inhibitory functions for uPA and plasmin (109, 110).

In addition to serpin-dependent regulation of fibrinolysis, there are other non-serpin regulators/inhibitors of fibrinolysis. Thrombin-activatable fibrinolysis inhibitor (TAFI; also called carboxypeptidase B2, CPB2) is a non-serpin negative inhibitor of plasmin activity and acts as the terminal enzyme of the thrombotic cascade. TAFI is synthesized by the liver and megakaryocytes as an inactive zymogen (111, 112) and is processed to the functional form by thrombin, the thrombin-thrombomodulin complex, or plasmin (113–115). TAFI is secreted at circulating concentrations of 4–15 μg/mL (116). Processed TAFI exerts its anti-fibrinolytic effect by cleaving the C-terminal lysine residues which act as the plasminogen-binding site mediating plasminogen-to-plasmin conversion (117). Thus, active TAFI reduces fibrinolysis by suppressing the *in situ* activation of plasmin.

Alpha-2-macroglobulin (A2M) is a large (720 kDa) broad-spectrum inhibitor of an expansive array of proteases across all catalytic classes, including trypsin, chymotrypsin, thrombin, plasmin, kallikrein, uPA, cathepsins, papain, and matrix metalloproteinases among others (118). Accordingly, A2M can act as an inhibitor of both thrombosis and fibrinolysis. A2M is primarily synthesized in the liver, but *in vitro* experiments indicate that cultured cells from the lung as well as macrophages and microglia can also synthesize and secrete A2M (119–121). In the fibrinolytic cascade, A2M inhibits the activity of plasmin and its upstream activator uPA (122).

## Fibrinolysis Pathway-Associated Serpins as Therapeutics in Inflammatory Disease

The dysregulation of fibrinolytic signaling is now identified as an important component of numerous pathologies. Genetic deficiencies in fibrinolytic regulation lead to bleeding disorders, organ dysfunction, and damage, and acquired disorders cause tissue fibrosis, dysregulated bleeding, cirrhosis, amyloidosis, and certain cancers (123). Several recent reviews discuss the role of the fibrinolytic system in inflammation and the immune response (124, 125). Similarly, small molecules inhibitors of fibrinolytic serine proteases such as tranexamic acid are under investigation for therapeutic modulation of the fibrinolytic processes that are associated with effects on the immune response (126). However, the highly evolved and potent inhibitory mechanisms of serpins have led to a growing interest in using serpins themselves as therapeutics. In the following section, we will discuss examples of the therapeutic use of fibrinolysis pathway-associated serpins, limiting our overview to examples which have been tested in preclinical models.

### PAI-1: Therapeutic Applications

Considering the delicate balance of the canonical targets of PAI-1—tPA and uPA—and the detrimental consequences of their dysregulation after injury, PAI-1 is a natural choice for therapeutic modulation of fibrinolysis dysregulation and has been demonstrated as such in numerous studies. PAI-1 is frequently seen as a mediator of injury and has been experimentally targeted to limit disease, especially cancer (127). The use of PAI-1 as a therapeutic at first may be unexpected. A large portion of studies investigating the delivery of PAI-1 for therapeutic purposes center on the cardiovascular system where thrombotic occlusion of infarction is treated with tPA, a target for PAI-1. However, PAI-1 has been extensively studied in numerous preclinical non-thrombotic animal disease models with demonstrated benefit. Unexpectedly, Carmeliet et al. demonstrated that adenovirus-mediated gene transfer of human PAI-1 in mice prior to induction of electric or mechanical vascular injury of the femoral or carotid arteries, respectively, reduced arterial neointima formation, a precursor to occlusive arterial plaque (128). This finding was supported by work from Schäfer et al. showing that bone marrow-derived PAI-1 reduces neointimal formation and luminal stenosis in bone marrow transplantation after carotid injury with ferric chloride (129). In subsequent work, Wu et al. demonstrated that recombinant PAI-1 prevented intimal hyperplasia in a model of carotid artery injury in rats, thus further supporting a potential therapeutic role for PAI-1 to prevent vascular restenosis (130). Interestingly, their systematic investigation involving constitutively active, inhibition-defective, and vitronectin binding-deficient forms of PAI-1 demonstrated that the ability to therapeutically limit intimal hyperplasia was mediated by either the ability for the serpin to inhibit proteases, or to bind to vitronectin, but did not require both. Zhong et al. similarly demonstrated that recombinant PAI-1, differentially through binding to vitronectin or protease inhibitory activity, mediates a therapeutic reduction of cardiac fibrosis in a model of cardiac fibrosis in uni-nephrectomized mice fed a high salt diet and angiotensin II (131). Using an adenovirus-5 (Ad5) with a CMV promoter, Qian et al. found that overexpression of human PAI-1 protected ApoE-deficient mice from abdominal aortic aneurysm induced by angiotensin II when delivered directly into the perivascular tissue of the aorta, but not when delivered systemically by tail-vein injection (132). Ad5-mediated gene transfer of human PAI-1 was also shown by Heymans et al. to preserve pump function in mice after acute pressure overload by attenuation of left ventricular remodeling (129). In a highly rational translation of its natural function, Jankun et al. reported that a modified version of PAI-1 with a half-life increased from 2 h to more than 700 h (very long half-life, termed VLHL PAI-1) was therapeutically effective at promoting hemostasis in reducing total blood loss induced by tail clipping when given by systemic or topical administration in mice (133).

PAI-1 has also demonstrated therapeutic efficacy in models outside of the conventional cardiovascular system. Yang et al. identified a role for tPA in the breakdown of the blood-brain barrier during neonatal cerebral hypoxia-ischemia in rats (134). Administration of recombinant PAI-1 dose-dependently preserved brain tissue and reduced edema, axonal degeneration and cortical cell death. The same group further adapted the treatment to an intranasal delivery format, which reached the cerebral cortex and reduced ~75% of brain atrophy in hypoxic-ischemic brain injury of newborns and in lipopolysaccharide-sensitized hypoxic-ischemic brain injury (135). Swiercz et al. demonstrated that exogenous delivery of the 14-1b active mutant of PAI-1 with an extended half-life limited angiogenesis and LNCaP prostate cancer tumor growth in SCID mouse xenografts when delivered by continuous infusion with subcutaneously implanted osmotic pumps (136). Praus et al. reported that liver adenoviral delivery of PAI-2, but not PAI-1, reduced the incidence of lung and brain liver tumor metastases but did not increase mouse survival (137).

PAI-1 therapy may also have a role in treating non-sterile conditions, as the pro-inflammatory functions of PAI-1 are found to be critical in numerous animal models of difficult-to-treat infections (138–140). Renckens et al. identified an essential role for PAI-1 in the host response to the respiratory pathogen *Klebsiella pneumoniae*, a Gram-negative bacteria, demonstrating an enhanced immune response, reduced lethality and prevention of sepsis and distal organ injury in mice transgenically overexpressing human PAI-1 delivered by Ad5 vector (141). Interestingly, the authors found that intranasal Ad5 delivery of PAI-1, but not Ad5 delivery alone in healthy mice also induced pulmonary inflammation and suggested that increased PAI-1 levels in the lungs may prime a protective inflammatory response in the context of infection.

### PN-1: Therapeutic Applications

PN-1 is expressed by many tissues in the body and thus exhibits broad potential regulatory functions outside of a role in modulating fibrinolysis in the circulation. Several features of PN-1 function have been translated to a potential for therapeutic development. Activation of fibrinolytic machinery, such as uPA and tPA, in tissues activates downstream matrix metalloproteinases (MMPs), leading to degradation of collagen and elastin in the extracellular matrix architecture (142). Stevens et al. demonstrated that intraarticular administration of recombinant PN-1 prevents articular cartilage degradation in rabbits subjected to interleukin-1 beta/basic fibroblast growth factor insult (143). Curiously, McKee et al. discovered that the PN-1 can engage the canonical serpin-enzyme complex receptor, lipoprotein receptor-related protein-1 (LRP-1), in the form of a PN-1:uPA complex to downregulate the activity of the sonic hedgehog (SHH) pathway which is involved in the malignant transformation of numerous tissues (144). Treatment of PC3 prostate cancer cells with recombinant PN-1 or combinatorial treatment with SHH pathway inhibitors significantly reduced xenograft tumor growth in SCID mice and was associated with alterations in angiogenesis (144). In a subsequent study, the same group identified that PN-1 may act via inhibition of X-chromsome-linked inhibitor of apoptosis (XIAP) and reported that therapeutic treatment of xenograft tumor growth in SCID mice was also synergistic with XIAP inhibitors (145). A potential limitation of both of these studies, however, is the use of a pre-treatment regimen for investigation. It will be valuable to know whether PN-1 has anti-tumor activity after tumor engraftment and growth. The utility of PN-1-mediated targeting of the SHH pathway was recently demonstrated by Li and Wang et al. using a model of Alzheimer's disease in APP/PS1 transgenic mice (146). Hippocampal delivery of lentivirus particles to overexpress PN-1 resulted in improved cognitive function, reduced amyloid deposition and preserved neuronal cell viability.

### Alpha-2-Antiplasmin (A2AP): Therapeutic Applications

Owing to the fact that alpha-2-antiplasmin (A2AP) is the primary inhibitor of plasmin, the majority of research on A2AP in the therapeutic context has focused on diminishing the inhibitor activity of A2AP, thereby enhancing fibrinolysis (147). In contrast, less focus has been spent on investigating A2AP to limit bleeding. In early work, Weitz et al. found that supplementation with A2AP inhibited tPA-induced fibrinogenolysis and bleeding, but did not affect thrombolysis in a model of jugular vein thrombosis in rabbits (148). Nieuwenhuizen et al. investigated the therapeutic administration of A2AP in a model of joint bleeding-induced arthropathy, which can persist even after the administration of clotting factor (149). Using a model of needle-induced arthropathy in Factor VIII-deficient mice, the authors found a reduction in both synovitis and cartilage damage over a period of 5 weeks when antiplasmin was given by direct intraarticular administration, whereas the uPA inhibitor amiloride was ineffective (149, 150). A2AP has also been found to have efficacy in limiting cancer burden. Hayashido et al. reported a drastic reduction of SCCKN squamous cell carcinoma tumor growth in SCID mice when the cells overexpressed A2AP vs. a mock-expressing control. A2AP-mediated reductions in tumor growth occurred by limiting E-cadherin processing by the fibrinolysis pathway (151). Similarly, Paquet-Fifield et al. reported restricted lymphatic remodeling and reduced metastases in SCID mice harboring 293-EBNA cells overexpressing A2AP and VEGF-D vs. LacZ (152). A limitation in these studies investigating the role of A2AP on tumorigenesis is again the prior delivery of A2AP to cells before implantation. Investigations determining if post-implantation delivery of the A2AP gene sequence or recombinant protein will have a similar effects could advance A2AP as a therapeutic.

### Neuroserpin: Therapeutic Applications

The therapeutic use of neuroserpin has primarily been investigated in neuropathologies. In early studies Yepes et al. found that intracerebral administration of neuroserpin was protective after in a model of middle cerebral artery occlusion (MCAO) stroke and prevented basement membrane proteolysis and cellular apoptosis in the ischemic penumbra of rats by more than 50% (153). In subsequent work, Zhang et al. used neuroserpin as an adjuvant treatment and found that intracisternally-injected neuroserpin increased the therapeutic window for therapeutic tPA administration after MCAO in rats by as much as 4 h with reduced brain edema and ischemic lesion volume (154). This work was recently confirmed in a similar study by Cai et al. (155). Interestingly, Wu et al. showed that the neuroprotective effect of neuroserpin in experimental MCAO was independent of its ability to inhibit tPA because protection was observed even in tPA-deficient mice, suggesting broader protective mechanisms in cerebral ischemia potentially involving less efficiently-inhibited serine proteases such as plasmin (156). Yepes et al. further reported that administration of neuroserpin into the ipsilateral hippocampus enhances neuronal survival and delays the progression of seizure activity in rats and mice subjected to kainic acid-induced seizures (157). Labeurrier et al. also showed neuroprotection against NMDA-induced excitotoxicity when neuroserpin was co-injected with NMDA into the left striatum or left cortex of mice (158).

Leveraging the neuroserpin therapeutic benefits on brain-associated pathologies, several studies have expanded investigations into the broader therapeutic effects of neuroserpin. In a rat model of spinal cord injury induced by clip compression, neuroserpin immediately injected intrathecally increased numbers of anterior horn motor neurons associated with restoration of autophagy and improved functional recovery as determined by the Basso Beattie Bresnahan scoring system (159). Upon intravitreal administration, neuroserpin protected against retinal ischemia-reperfusion injury induced by elevated intraocular pressure associated with attenuation of apoptosis (160). In other studies examining neuroserpin as an immune modulating therapeutic, Munuswamy-Ramanujam et al. reported that intravenous administration of recombinant neuroserpin prevented vasculopathy in a mouse aortic allograft transplant model. In this model, neuroserpin reduced plaque growth and T-cell invasion and T helper cell responses (161). In contrast, neuroserpin was ineffective when evaluated as a treatment for severe gammaherpesviral (MHV68) infection and associated vasculitis in interferon gamma receptor-deficient (IFNγR^−/−^) mice (162). The highly specific endogenous sequestration of neuroserpin to the central nervous system may provide certain advantages to its therapeutic administration as it may not be subjected to the same degree of negative regulation in extra-neural tissues. While currently under-explored, studies investigating neuroserpin efficacy in other serpin-sensitive therapeutic scenarios are warranted.

## Alpha-1-Antitrypsin: A Promiscuous Serpin With Potent Therapeutic Properties

Alpha-1-antitrypsin (A1AT or AAT, SERPINA1) is the prototypical and best studied member of the serpin superfamily (163). While neutrophil elastase is the prominent and most characterized target of A1AT, leading to a reduction in neutrophilic inflammation (164), early investigations of A1AT activity identified a broad serine protease reactivity with cathepsins, caspases, metalloproteases, and coagulation cascade-associated serine proteases thrombin and plasmin (165). Interestingly, Talens et al. reported that A1AT is the most abundant non-covalently bound protein in fibrin clots and remains functionally active as a serpin *in situ* (166). The exclusion of A1AT from classical descriptions of fibrinolytic regulation may thus be an oversight, due to a focus on current understanding of therapeutic benefit in lung disease and underrepresenting the local control of plasmin activity by A1AT directly in the fibrin clot.

A1AT deficiency is a potentially severe, chronic condition characterized by unregulated inflammation primarily in the lungs, leading to COPD and emphysema, and in the liver leading to cirrhosis. Given A1AT potency as an inhibitor of serine proteinases, A1AT deficiencies may also be associated with other under recognized complications, such as during post-surgical healing (167, 168). A1AT recombinant protein therapy (augmentation therapy) is clinically eficacious, thereby prompting A1AT gene therapy, systemically administered via viral vectors, to advance into clinical trials and yield promising results (169).

Beyond treatment of serpin genetic deficiencies, A1AT is a broad and potent immune modulator. In early work, Libert et al. demonstrated a protective role for A1AT, which they first identified as an acute phase reactant, in lethal TNF insult in mice by a mechanism dependent on reducing platelet-activating factor and associated with reversals of body temperature drop, liver injury and increased clotting time when given recombinantly by intraperitoneal or intravenous administration (170). Later, the therapeutic effect of A1AT was demonstrated by Churg et al. in a model of cigarette smoke-induced emphysema in mice, which the authors suggested may be related to inhibition of both matrix metalloproteinases as well as TNF signaling (171). The protective effect of A1AT in the lungs may underscore the distinct physiological role observed with genetic deficiency. Similarly, Wang et al. found that A1AT treatment limited pulmonary apoptosis and necrosis in a rat model of ventilator-associated acute respiratory distress syndrome (ARDS) (172). Akbar et al. found that gene therapy with A1AT delivered by adeno-associated virus-8 (rAAV8) ameliorated bone loss in an ovariectomy-induced osteoporosis mouse model of post-menopause osteoporosis which was associated with inhibition of IL-6 and RANK levels (173).

A1AT has demonstrated repeated therapeutic efficacy in various models of cellular transplantation. Lewis et al. reported pancreatic islet transplantation survival was extended by treatment with recombinant clinical-grade human A1AT, associated with a reduction of inflammatory cell infiltration and abrogation of TNF signaling (174). This effect was extended to preservation of islet cell viability in streptozotocin-treated mice. In a related study, Zhang et al. described that A1AT-dependent protection of islet viability after cytokine- and streptozotocin-induced diabetes in mice was in part due to dramatic reduction of beta cell apoptosis (175). Recently, the protective effect of A1AT in islet cell transplantation was demonstrated by Gou et al. in an intrahepatic transplant model in NOD-SCID mice by suppressing macrophage activation with reduced TNF, iNOS, IL-6, and CD11c signals (176). A1AT has been effective in preventing graft rejection in other cellular transplant models, as well. Marcondes et al. showed that A1AT prevented graft-vs.-host disease (GVHD) in an allogeneic murine transplantation model in both a preconditioning and post-conditioning treatment regimen (177). In a similar follow-up study, Tawara et al. confirmed the protective effect of A1AT in bone marrow transplant GVHD and described an associated reduction in TNF, IL1b, IL-6, and NF-kappaB signaling (178). Lee et al. showed that intravenous administration of A1AT reduced short-term engraftment of hepatocytes in rats which remained significant at 48 h (179). While significance was lost at longer timepoints, there was evidence of viable engrafted hepatocytes up to 1 month after transplant, which was not apparent in control mice. Recent work by Emtiazjoo et al. showed a remarkable reduction of acute lung allograft injury in an orthotopic single left lung transplantation model from Lewis to Sprague-Dawley rats at 8 days post-transplantation (180). Of crucial importance, protection was achieved in the absence of any systemic immunosuppression.

Addressing another complication of diabetes, Ortiz et al. reported that A1AT treatment alleviated the progression of diabetic retinopathy in mice by suppressing TNF signaling in both the serum and retina and promoting an M2-polarized macrophage population, which ultimately delayed ganglion cell loss and retinal thinning (181). In another study examining A1AT efficacy for ophthalmological disorders, Yang et al. demonstrated protection of iPSC grafts after subretinal transplantation into the eye of mice with preexisting ocular hypertension by inhibition of microglial activation (182). Interestingly, Zhou et al. reported that suppression of microglial inflammation and neurodegeneration in the eye was achieved by intraperitoneal injection of A1AT in a Rd1(FVB/N) mouse model of retinal degeneration (183).

Ischemia-reperfusion injury is characterized by a transient loss of blood and oxygen to a tissue, followed by a period of reoxygenation which paradoxically accelerates damage caused during the hypoxic period (184). Ischemia-reperfusion injury can occur in any tissue, whether by pathogenic etiology or by complications of surgical procedure, and there is an unmet need for novel therapeutics to address the condition (185–188). Moldthan et al. first demonstrated the therapeutic efficacy of A1AT therapy in a rat model of ischemic stroke which resulted in a drastic reduction of infarct volume and preservation of sensory motor system function (189). Toldo et al. generated a recombinant A1AT-Fc fusion protein and demonstrated efficacy in reducing inflammation following myocardial ischemia-reperfusion in mice which they found was independent of the capacity to inhibit elastase (190). In translation of this work, the VCU-a1RT clinical trial (NCT01936896) was undertaken to investigate the potential protective effect of A1AT therapy (Prolastin^®^) in patients with ST-segment elevation myocardial infarction (STEMI) (191). Abbate et al. reported that the VCU-a1RT trial found no in-hospital adverse effects of A1AT therapy and that a blunted initial inflammatory response resulted in significantly reduced CRP levels 14 days after admission. In further analysis of the trial, Abouzaki et al. also described a shorter time-to-peak in CK-MB levels indicating an inhibition of the onset of inflammatory injury (192). In investigations of other tissues, Maicas et al. found a limited therapeutic efficacy for clinical grade human A1AT (Prolastin^®^) in a mouse model of renal ischemia-reperfusion injury where they reported a significant decrease in kidney injury molecule-1 levels in urine but no effect on renal fibrosis (193). This finding contrasts later work by Jeong et al. who reported a significant protection against renal ischemia-reperfusion injury upon treatment with A1AT, including attenuated tubular injury and fibrosis (194). The design of these two contrasting studies are similar in the use of FDA-approved clinical grade A1AT at a dose of 80 mg/kg/day, however Jeong et al. administrated A1AT for 3 days prior to surgery and only followed up at 24 h post-procedure, whereas Maicas et al. first administrated A1AT at 24 h pre-procedure and followed up at 8 and 15 days post-procedure. Thus, the fibrotic phenotype likely developed over a longer time course than observed by Jeong et al.

Lupus is an autoimmune condition characterized by dysregulated adaptive and innate immune responses in tissues and the vasculature which can have damaging and potentially lethal effects on end organs such as the kidneys and lungs (195). Elshikha et al. investigated the protective effects of A1AT therapy in a series of preclinical studies. In the first of the series, they showed that A1AT inhibits plasmacytoid dendritic cell activation and protects against nephritis in the MRL/lpr spontaneous lupus model (196). They went on to describe that gene therapy with A1AT delivered by rAAV8 prolongs lifespan in NZM2410 mice which develop spontaneous lupus with early-onset glomerulonephritis and that the protection was associated with reduced autoantibody levels (197). Recently, Elshikha et al. described that treatment with recombinant A1AT limited disease progression and suppressed TNF signaling in a pristane-induced model of acute lupus diffuse alveolar lung hemorrhage (DAH) (198). Thus, the broad immune modulating effects of A1AT highlight the significant therapeutic potential of this serpin with broad activity against a wide range of serine proteases in diverse diseases driven by dysregulated inflammatory processes.

## Serp-1: A Virus-Derived Coagulation Regulator and Therapeutic Immune Modulator

Viruses, especially DNA viruses with large genomes such as poxviruses and herpesviruses, have expertly evolved highly effective and potent immune modulating protein machinery that evade host immune defenses (199). These proteins have become the focus of a growing field of research around the development of virus-derived therapeutics (200), some of which have led to increased interest in mammalian serpin therapeutics. The most thoroughly investigated virus-derived therapeutic protein is Serp-1, a serpin from Myxoma virus which targets the fibrinolytic serine proteases uPA, tPA, and plasmin as well as the thrombotic proteases FXa and thrombin (in the presence of heparin) (201, 202). The first demonstration of Serp-1 therapeutic efficacy was in a rabbit model of aortic balloon angioplasty injury, where protection was characterized by significantly reduced inflammation and plaque growth (203). The doses used in that original study were single intravenous injections in the *picogram* range given immediately after angioplasty injury. This therapeutic effect against inflammation and vasculopathy with plaque growth was further demonstrated in models of aortic, renal and heterotopic heart transplants in mice and rats and in a mouse carotid compression model (204–207). Serp-1 was also demonstrated to be protective in a collagen-induced arthritis model in rats, with generalized immune modulation outside of transplant and pro-atherogenic disease states (208). In more recent work, Serp-1 was found to be an effective therapeutic against severe vasculitis in both human temporal artery biopsy transplants from patients suspected to have Giant cell arteritis into SCID mice and in the lethal MHV68 gammaherpesvirus-induced vasculitis in interferon gamma receptor-deficient mice (162, 209). Interestingly, peptides derived from Serp-1 are also therapeutically effective in the gammaherpesvirus-induced vasculitis model and protection imparted by both the full protein and the peptide derivatives are dependent on composition of the gut microbiome (202, 210, 211). *In vitro* studies demonstrated that, these reactive center loop (RCL) peptides bound and inhibited mammalian serpins (202). While these studies all used an intraperitoneal or intravenous delivery of naked recombinant protein, Serp-1 is also amenable to drug delivery vehicles and is currently the only serpin demonstrated for delivery by such approaches. Serp-1 is capable of sustained delivery in a chitosan-collagen biocompatible hydrogel and has demonstrated therapeutic efficacy in models of full-thickness cutaneous wound healing in mice and in spinal cord injury in rats, with efficacy dependent on engagement of uPAR in the fibrinolytic signaling pathway (212–214).

Serp-1 is a *First-in-Class* therapeutic and the first virus-derived protein given to humans in an FDA-overseen Phase IIa clinical trial (NCT00243308) for patients with unstable coronary syndromes, unstable angina and small heart attacks (215). Serp-1 was safe and well-tolerated with a major adverse cardiac event score (MACE) of zero and with a dose-dependent reduction in heart damage markers Troponin and CK-MB. Importantly, there were no detectable neutralizing antibodies against Serp-1. Thus, Serp-1 represents a cross-pathway serpin targeting thrombolytic and thrombotic cascades which regulates inflammatory responses in part by engaging signaling in the fibrinolytic pathway with potent therapeutic efficacy in a wide variety of disease states.

## Fibrinolysis and Serpins in SARS-CoV-2 Infection and the COVID-19 Pandemic

Understanding the bidirectional activation of the coagulation proteases and activation of immune responses indicates a clear target for serpin therapeutics in severe infections where both coagulopathy as well as excessive and damaging immune response cause increased damage and mortality. Serpins have been examined in preclinical models of severe viral infections with coagulopathy, one notable example being the use of Serp-1 treatment in the severe vasculitis/lung hemorrhage model in IFNγR^−/−^ mouse models. In these models, as noted, Serp-1 improves survival and reduces both lung consolidation as well as vascular inflammation. Similarly, PAI-1 has proven beneficial in models of severe Klebsiella pneumoniae. Antithrombin III (ATIII), a serpin inhibiting the clotting pathway and activated by heparin infusions, also has had variable benefit in clinical trials of bacterial sepsis in man.

In December 2019, a pneumonia of unknown origin was identified in Wuhan, the capital of Hubei province in China and identified as a Severe acute respiratory distress syndrome (SARS) coronavirus denoted as SARS-CoV-2 (216). The disease caused by SARS-CoV-2, is referred to as coronavirus disease 2019 (COVID-19) and is now a worldwide pandemic as declared by the World Health Organization (217). As of March 10th, 2021, >118 million cases of COVID-19 has been reported globally with a death toll >2.6 million worldwide (Worldmeters.info).

Symptomatically, COVID-19 commonly causes severe coughing and hemoptysis, shortness of breath and hypoxemia accompanied by widespread lung infiltrates, consolidation and in some cases hemorrhage, with fever, weakness and confusion. SARS-CoV-2 is therefore identified, along with SARS-CoV-1, as a severe acute respiratory disease (218). However, despite the commonality of ARDS in COVID-19 patients, mounting evidence suggests that infection with SARS-CoV-2 induces a hypercoagulable state (219). Spiezia et al. reported that severe hypercoagulability, but not a clearly defined consumptive coagulopathy (or disseminated intravascular coagulopathy, DIC), is present in COVID-19 patients with acute respiratory failure (220). Endothelial injury and dysfunctional coagulation, with associated resistance against fibrinolysis, may thus reclassify COVID-19 as a vascular disorder complicated by widespread microthrombotic occlusions rather than a respiratory disease (221). We direct readers to a thorough description of this hypothesis in a recent review by Siddiqi, Libby and Ridker (222).

The apparent dysfunction of fibrinolysis during COVID-19 infection would suggest that therapeutic administration of serpins of the fibrinolytic pathway against SARS-CoV-2 may not be beneficial. However, many factors are worth consideration in the use of serpins in severe viral septic states with imbalance in coagulation as well as excess aggressive immune responses in accompanying cytokine storm. First, on a molecular level, SARS-CoV-2, like other coronaviruses, requires proteolytic processing of its Spike (S) protein in order to appropriately dock with and enter host cells (223, 224). It is now known that the SARS-CoV-2 S protein is processed sequentially by the subtilisin-like peptidase furin and the transmembrane serine protease TMPRSS2 (225). Indeed, mechanistic studies have shown that a clinically approved TMPRSS2 inhibitor, camostat mesylate, inhibited SARS-CoV-2 S-driven infection *in vitro* (226). A recent retrospective observational case series on a small cohort of ICU patients in Germany found reduced severity in patients treated with camostat mesylate vs. those who received hydroxychloroquine (227).

**Figure 4:**
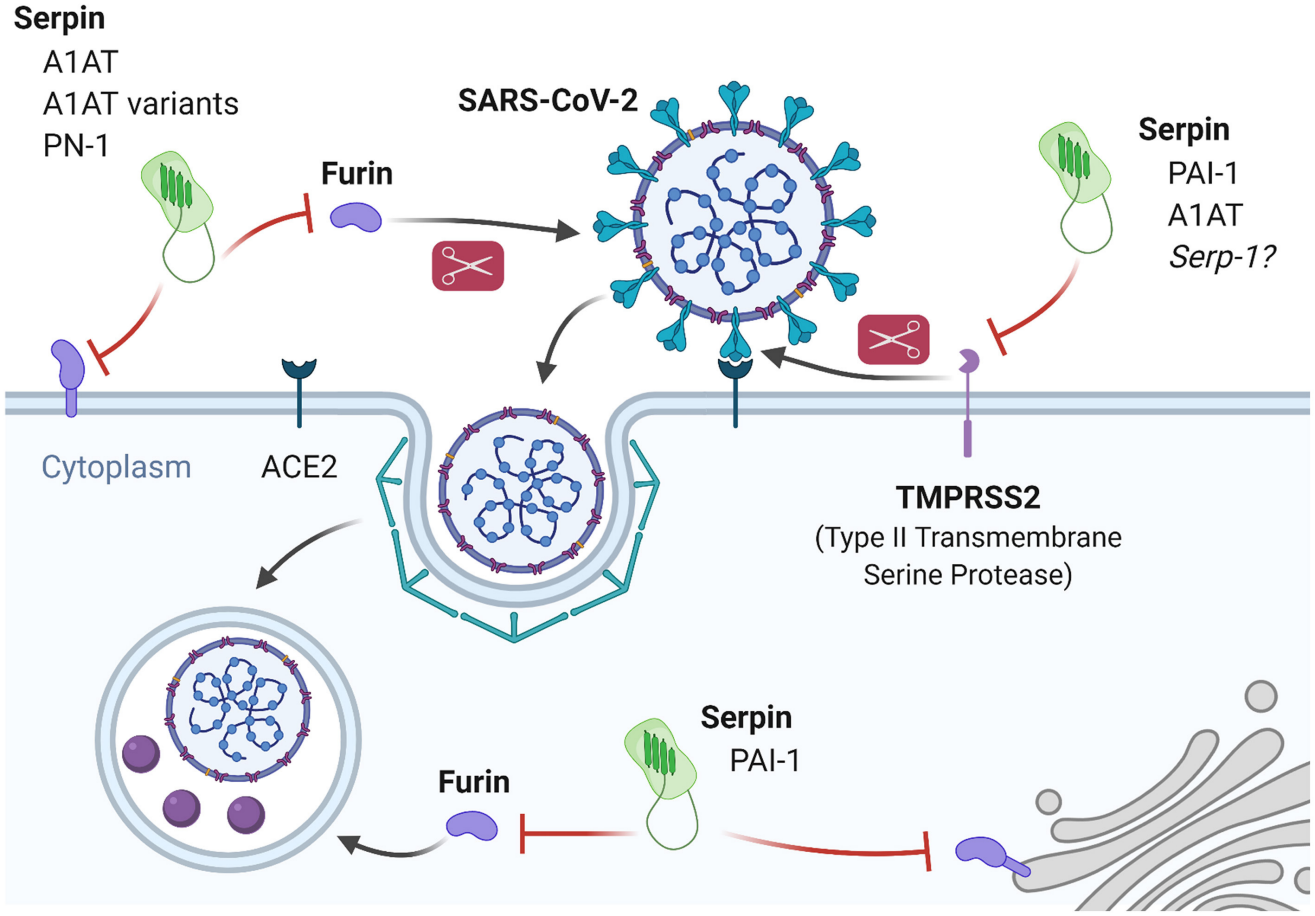
Inhibition of SARS-CoV-2 infection and processing by fibrinolysis pathway-associated serpins. SARS-CoV-2 entry is dependent on proteolytic processing of the spike protein in order to engage the ACE2 receptor and internalize. Processing of the spike protein is performed primarily by TMPRSS2 and Furin. PAI-1 and A1AT are known inhibitors of TMPRSS2 and experimental evidence demonstrates that A1AT can inhibit SARS-CoV-2 infection *in vitro*. Based on similarities in inhibition properties, it may be predicted that Serp-1 will also inhibit TMPRSS2 with similar results. Furin has both an extracellular and intracellular role in the SARS-CoV-2 life cycle and there is evidence for extracellular furin inhibition by A1AT and A1AT variants (Portland and α-PDX) as well as endothelial PN-1, and for intracellular furin inhibition by PAI-1.

Several fibrinolysis pathway serpins may interfere with these mechanisms (Figure 4). A preprint by Azouz et al., first deposited in May 2020, demonstrated that A1AT inhibits TMPRSS2 in an HEK-293T overexpression system (228). In an important follow-up study deposited in July 2020, where Wettstein et al. produced SARS-CoV-2 S-protein pseudoparticles and performed *in vitro* infection of Caco2 cells in the presence of chromatographically fractioned bronchoalveolar lavage samples they found that the highest inhibition of infection occurred in the fraction containing A1AT (229). These findings agree with prior reports of the ability for A1AT to inhibit infectivity of TMPRSS2-dependent viruses. Beard et al. reported that A1AT inhibits *in vitro* and *in vivo* mouse infection by H1N1 Influenza, which requires hemagglutinin processing via TMPRSS2 (230). Similarly, Esumi et al. reported that hepatitis C virus infection proceeds by the activity of TMPRSS2, which was dose-dependently inhibited *in vitro* by A1AT (231).

Second, on a coagulation systemic level, serpins target areas with activated serine proteases. Thus serpins are predicted to target areas with active thrombosis and or thrombolysis. Dysregulated thrombosis and thrombolysis are now clearly an important component of COVID-19 disease (232). What is not clear is how or why these pathways become dysregulated, and some have proposed that increased amounts of active PAI-1 may induce a feed-forward loop of inflammatory events (233). This idea has led to the recent initiation of a clinical trial to test the PAI-1 inhibitor, TM5614, for treating high-risk patients hospitalized with severe COVID-19 (NCT04634799). Similarly, tranexamic acid, a uPA inhibitor, is under investigation to combat COVID-19 (NCT04338074, NCT04338126). In further support of this, several groups have suggested modulation of the fibrinolytic pathway by targeting plasmin/plasminogen based on imbalances in protease levels in COVID-19 patients (234, 235). However, sensitivity in the pathway imbalance urges caution in administration, and the dynamics of the disease may dictate appropriate timing for intervention (236, 237).

While it is firmly established that coagulopathy occurs in COVID-19 patients, there is a possibility that PAI-1 may in fact be a protective host factor against SARS-CoV-2. Dittmann et al. reported that PAI-1 can dose-dependently inhibit Influenza A infection by preventing hemagglutinin processing by TMPRSS2 (238). Thus, the ability for PAI-1 to inhibit TMPRSS2 suggests that the problem of dysregulation of fibrinolysis may be more complex than focusing on PAI-1 may solve. Colling and Kanthi propose that the ratios of active PAI-1 and tPA may be more indicative of the pathway activity in COVID-19 patients due to ongoing consumption and microvascular thromboses (239). In support of this hypothesis, a recent preprint by Zuo et al. on 118 hospitalized COVID-19 patients and 30 healthy controls found that patients who died had significantly higher levels of both PAI-1 and tPA (240). Importantly, the authors found that a higher ratio of tPA vs. PAI-1 was indicative of potential mortality, and was driven by an increase in tPA, not PAI-1.

Furin is a second protease involved in S-protein priming during SARS-CoV-2 infection, and also has a role in intracellular processing (241). Furin is present in both membrane-bound and secreted, soluble states with the latter usually associated with a variety of pathologies, such as diabetes or infection (242–244). Cheng et al. recently reported that small molecule inhibitors of furin prevent SARS-CoV-2 infection as well as intracellular processing *in vitro* (245). However, the significant role of furin in normal tissue development and homeostasis make it a difficult target for therapeutic modulation and there are no FDA-approved furin inhibitors for clinical use. On the other hand, there is precedent for experimentally targeting furin with serpins to limit viral infection. For example, Shapiro et al. reported A1AT-mediated inhibition of HIV infection *in vitro*, which is dependent on furin-mediated processing of the membrane protein gp160 (246, 247). Numerous groups have engineered A1AT to fine-tune its properties, such as the A1AT Portland variant with increased specific and activity against furin described by Jean et al. to have anti-pathogenic properties (248). Similarly, Anderson et al. reported another A1AT variant (α1-PDX) with 3,000-fold higher anti-furin activity which potently inhibited HIV gp160-dependent infection *in vitro* (249). Furin is also inhibited by PAI-1 (intracellular furin) and endothelial PN-1 (extracellular furin), but their ability to limit infection via furin inhibition-dependent mechanisms remains to be explored (250, 251).

Third, the now understood evolution of serine proteases and serpins as regulators of both thrombosis and thrombolysis, and also of inflammation, would suggest a potential for the use of serpins that target both coagulation as well as immune responses in severe viral infections. The immune system response to SARS-CoV-2 reveals a different perspective for COVID-19 and fibrinolysis. Numerous inflammatory pathologies are associated with increased circulating levels of soluble uPAR (suPAR), produced by the cleavage of the C-terminal glycosyl-phosphatidylinositol linker by phospholipases (252). Growing evidence has established suPAR as a useful diagnostic and prognostic indicator of severe, acute pathologies including sepsis (253). Based on similarities of COVID-19 complications with diseases associated with or exacerbated by elevated suPAR, D'Alonzo et al. proposed suPAR as a therapeutic target for treating SARS-CoV-2 infection (254). In support of this proposition, early in the COVID-19 pandemic Rovina et al. identified elevated suPAR in 57 Greek patients and 15 American patients as a highly significant early prognostic indicator of severe outcomes in SARS-CoV-2 infection (255). More recently, Azam et al. investigated the association of acute kidney injury (AKI) in COVID-19 patients with suPAR (256). AKI occurs in up to 50% of severe COVID-19 patients and significantly increases morbidity and mortality. Azam et al. found that the highest tertile of suPAR levels was associated with a more than nine-fold increase in AKI in COVID-19 patients and was independent of inflammatory markers or demographic subgroups. The uPAR system has other roles in SARS-CoV-2 infection in addition to suPAR elevation. Ly6E is a member of the uPAR family of proteins and a lymphocyte marker associated with immunological regulation and also recently associated with host responses to viral infection (257). Zhao et al. demonstrated that Ly6E restricts the entry of human coronaviruses in an ectopic expression model using both the common HCoV-O43 as well as SARS-CoV-2 (258). Importantly, Pfaender et al. performed crucial *in vivo* experiments in wildtype and Ly6E-deficient mice that revealed a critical Ly6E-dependent host defense against coronaviruses, including MERS-CoV, SARS-CoV, and SARS-CoV-2 (259). Mechanistic studies performed by the authors demonstrate that Ly6E prevents coronavirus entry into host cells by preventing S-protein-mediated membrane fusion. Of interest, the virus derived serpin that we have studied extensively, Serp-1, and also PAI-1 bind and block the uPA/uPAR complex (212, 213). With Serp-1 this leads to marked anti-inflammatory function. Thus, the uPA/uPAR/suPAR system represents an attractive therapeutic target for modulation to treat or limit the severity of immune disorders as well as potentially specific treatment for COVID-19 disease progression.

The role of serpins and serine proteases of the fibrinolytic system in COVID-19 is complex and investigations on the potential therapeutic modulation of these processes with natural, virus-derived or engineered serpins, expanding the consideration of these proteins beyond only regulation of the fibrinolytic system, may be a valuable pursuit as many of these modulators are already found to be safe and effective, and in some cases FDA-approved.

## Discussion

The ancient roots of clot formation and clot dissolution speaks to the necessary role these pathways play in protection of the host from excess thrombosis and thrombolysis to immune-based disorders. In simplest terms, protection against loss of blood (or hemolymph in the case of lower organisms) after traumatic injury is an essential component of survival. However, the greater understanding of the role of these pathways in host responses to infection point to a more complex role for the coagulation and fibrinolysis cascades.

Focusing on the fibrinolytic cascade, we have reviewed the diversity of serine protease and serpin control of this essential pathway. The complexity of the pathway also underscores how critical this pathway is to maintain a normal homeostatic balance: dysregulation may easily lead to disease and therefore redundant control has evolved to maintain homeostasis and preserve host viability. A striking consequence of the evolution of serpin regulators of fibrinolysis is their potency and general safety. As we have discussed, these factors have led to the investigation of fibrinolysis-associated serpins as therapeutics. However, despite the dense and growing body of work justifying the application of serpins as therapeutics there are few, if any, indications outside of augmentation therapy for A1AT deficiency and related lung and liver diseases which have received FDA approval. There is a growing need for novel therapeutics for inflammation-related disorders and naturally-evolved or improved, engineered serpins may provide higher potency and lower off-target selectivity than small molecule inhibitors. We would suggest that more attention should be given to the investigation of serpins as new potential therapeutics. This is particularly urgent in the context of severe cases of ARDS and microthrombotic vascular complications in the COVID-19 Pandemic (and potentially other viral pandemics), where serpins may provide a uniquely multifunctional role in modulating the host immune response as well as the virus life cycle toward improved outcomes.

## Author Contributions

JRY wrote the first draft of the manuscript and produced all figures. LZ, QG, SEH, and ARL revised the manuscript. All authors approved the final version of the manuscript.

## Conflict of Interest

JRY, LZ, and ARL are inventors on several patents and patent applications relating to the use of Myxoma virus Serp-1 as a therapeutic. ARL is a co-founder of a small spin-out biotechnology company, Serpass Biologics Inc. which is developing Serp-1 as a therapeutic. The remaining authors declare that the research was conducted in the absence of any commercial or financial relationships that could be construed as a potential conflict of interest.
